# Erratum

**DOI:** 10.4269/ajtmh.16-0579err

**Published:** 2018-02

In *Am J Trop Med Hyg 96(5):* 1066–1070 by Varghese and Al-Hajoj (https://doi.org/10.4269/ajtmh.16-0579), the financial support section of the paper is missing. It should read:

“This study has been funded by King Abdulaziz City for Science and Technology, Riyadh, Saudi Arabia under the National Science, Technology and Innovation Plan (NSTIP: Grant#11-BIO-1436-20).”

